# Paternal alcohol exposures program intergenerational hormetic effects on offspring fetoplacental growth

**DOI:** 10.3389/fcell.2022.930375

**Published:** 2022-08-11

**Authors:** Kara N. Thomas, Katherine N. Zimmel, Alison Basel, Alexis N. Roach, Nicole A. Mehta, Kelly R. Thomas, Luke J. Dotson, Yudhishtar S. Bedi, Michael C. Golding

**Affiliations:** Department of Veterinary Physiology and Pharmacology, College of Veterinary Medicine and Biomedical Sciences, Texas A&M University, College Station, TX, United States

**Keywords:** hormesis, alcohol, paternal, epigenetic programming, developmental programming, placenta, Fetal Alcohol Spectrum Disorder (FASDs), genomic imprinting

## Abstract

Hormesis refers to graded adaptive responses to harmful environmental stimuli where low-level toxicant exposures stimulate tissue growth and responsiveness while, in contrast, higher-level exposures induce toxicity. Although the intergenerational inheritance of programmed hormetic growth responses is described in plants and insects, researchers have yet to observe this phenomenon in mammals. Using a physiologically relevant mouse model, we demonstrate that chronic preconception paternal alcohol exposures program nonlinear, dose-dependent changes in offspring fetoplacental growth. Our studies identify an inverse j-shaped curve with a threshold of 2.4 g/Kg per day; below this threshold, paternal ethanol exposures induce programmed increases in placental growth, while doses exceeding this point yield comparative decreases in placental growth. In male offspring, higher paternal exposures induce dose-dependent increases in the placental labyrinth layer but do not impact fetal growth. In contrast, the placental hypertrophy induced by low-level paternal ethanol exposures associate with increased offspring crown-rump length, particularly in male offspring. Finally, alterations in placental physiology correlate with disruptions in both mitochondrial-encoded and imprinted gene expression. Understanding the influence of ethanol on the paternally-inherited epigenetic program and downstream hormetic responses in offspring growth may help explain the enormous variation observed in fetal alcohol spectrum disorder (FASD) phenotypes and incidence.

## Introduction

Fetal alcohol spectrum disorder (FASD) is a term that describes a broad range of presentations and disabilities attributed to the maternal consumption of alcohol during pregnancy (Cook et al., 2016). In the US, prevalence estimates range from 10 to 50 per 1,000 children, making FASDs the most predominant non-genetic birth defect associated with intellectual disability (Hoyme et al., 2016; May et al., 2018). Unfortunately, despite many years of study, we still lack a firm pathophysiological understanding of this condition and cannot fully explain the enormous variation in FASD phenotypes and prevalence (Schaefer and Deere, 2011; McCarthy and Eberhart, 2014).

While the risks of maternal alcohol use during pregnancy are well-established, emerging clinical studies indicate that paternal alcohol exposures also induce congenital disabilities and impact offspring behavior (Xia et al., 2018; Zhang et al., 2020; Luan et al., 2022). In support of these limited reports, recent preclinical studies reveal that paternal alcohol exposures negatively affect offspring phenotype and long-term health (Rompala and Homanics, 2019). For example, our group has identified short-term impacts of paternal alcohol exposure on fetoplacental growth and long-term influences on inflammation and glucose metabolism (Chang et al., 2017; Chang et al., 2019a; Chang et al., 2019b; Bedi et al., 2019; Thomas et al., 2021). Further, research by the Homanics and Huffman groups has independently associated preconception paternal alcohol exposures with blunted stress-related responses, altered preference for ethanol, abnormal sensorimotor activities, short-term motor learning impairments, and region-specific changes in gene expression within the brain (Finegersh and Homanics, 2014; Finegersh et al., 2015; Rompala et al., 2017; Beeler et al., 2019; Conner et al., 2020; Rathod et al., 2020). These later studies have profound implications for the heritability of alcohol use disorders, while collectively, this work demonstrates that chronic male alcohol exposure impacts offspring development and, therefore, must be considered when assessing FASD outcomes.

Our efforts to determine how preconception paternal alcohol exposures affect offspring phenotype have focused on non-genomic, epigenetic mechanisms of inheritance transmitted through sperm, including DNA methylation, histone posttranslational modifications, and small noncoding RNAs. Our studies examining alcohol-exposed sperm identified negligible changes in the genome-wide DNA methylation profile (Chang et al., 2017). However, the Homanics group and our lab have independently identified ethanol-induced changes in sperm noncoding RNAs (Rompala et al., 2018; Bedi et al., 2019; Rompala et al., 2020). Although correlative, these data demonstrate that chronic alcohol exposures induce epimutations in sperm, which may negatively influence the developmental program of the offspring. Nevertheless, many questions remain, such as how alcohol influences epigenetic mechanisms in the male reproductive tract, which phases of germline programming represent critical windows, or what levels of ethanol consumption elicit changes in epigenetic programming in sperm.

The question of dose is intriguing as many direct physiological responses to alcohol are J-shaped, meaning lower doses exert distinct outcomes from higher exposures. For example, clinical studies indicate alcohol has a hormetic influence on coronary heart disease and ischemic stroke, where lower consumption exerts protection beyond those who do not drink, while higher exposures enhance disease onset (Fernández-Solà, 2015). The term hormesis refers to the phenomena where low concentration toxicant exposures stimulate biological systems while higher concentrations inhibit these same processes, resulting in a biphasic response. Importantly, emerging research indicates that epigenetic mechanisms are partially responsible for hormetic programming and the beneficial effects of adaptive conditioning (Leak et al., 2018). Indeed, conditioning an ischemic-tolerant brain requires transcriptional repression by polycomb group (PcG) proteins, and suppressing this complex blocks the programmed gene expression underlying ischemic tolerance (Stapels et al., 2010). Although studies in insects, plants, and coral demonstrate the parental transmission of hormetic responses to their offspring (Kishimoto et al., 2017; Brevik et al., 2018; Putnam et al., 2020), there are no clear examples of intergenerational hormesis described using mammalian systems.

Our previous studies have correlated chronic paternal alcohol exposures with fetal growth restriction, placental overgrowth, and sex-specific changes in placental histology (Chang et al., 2017; Bedi et al., 2019; Thomas et al., 2021). Throughout these studies, we have consistently observed an impact of paternal drinking on the placental weights of male offspring. Therefore, using gestational day 16.5 placental weights within the F1 male offspring as a phenotypic marker, we set out to examine the ability of ethanol to induce dose-dependent effects on the paternally inherited epigenetic program.

We began our studies by defining a clinically relevant range of low and modest exposure levels based on previous works examining the impacts of alcohol on central nervous system function (Eckardt et al., 1998). These previous studies revealed that ethanol concentrations within the range of 0.7–2.0 g/kg reproducibly impacted CNS function in C57BL/6 mice. Therefore, we employed alcohol concentrations below, as well as at the lower and upper ends of this identified range. Using the previously determined average session fluid consumption of 0.017 g/g (Chang et al., 2019a), we administered concentrations (w/v) of 3% (0.5 g/kg, a concentration below the identified range), 6% (1.0 g/kg, lower end of the range), and 10% ethanol (1.7 g/kg, upper end of the range). We hypothesized that alcohol exposures falling within the established range would exert an intergenerational impact on placental growth, while the low concentration exposures below this established range would not. Unexpectedly, our studies demonstrate that paternal alcohol use programs nonlinear effects on fetoplacental growth, and significantly, even low dose alcohol exposures influence the sperm-inherited developmental program.

## Materials and methods

### Animal husbandry and preconception male alcohol exposures

We used C57BL/6J mice (Strain #:000664 RRID: IMSR_JAX:000664), which we derived from a breeder nucleus housed in the Texas A&M Institute for Genomic Medicine. We housed mice under a reverse 12-h light/dark cycle, with lights out at 8:30 a.m. and lights on at 8:30 p.m. We maintained mice on a standard chow diet (catalog# 2019, Teklad Diets, Madison, WI, United States). We implemented additional animal husbandry measures to minimize stress, including shelter tubes for males and igloos for females (catalog# K3322 and catalog# K3570, Bio-Serv, Flemington, NJ, United States).

**FIGURE 1:**
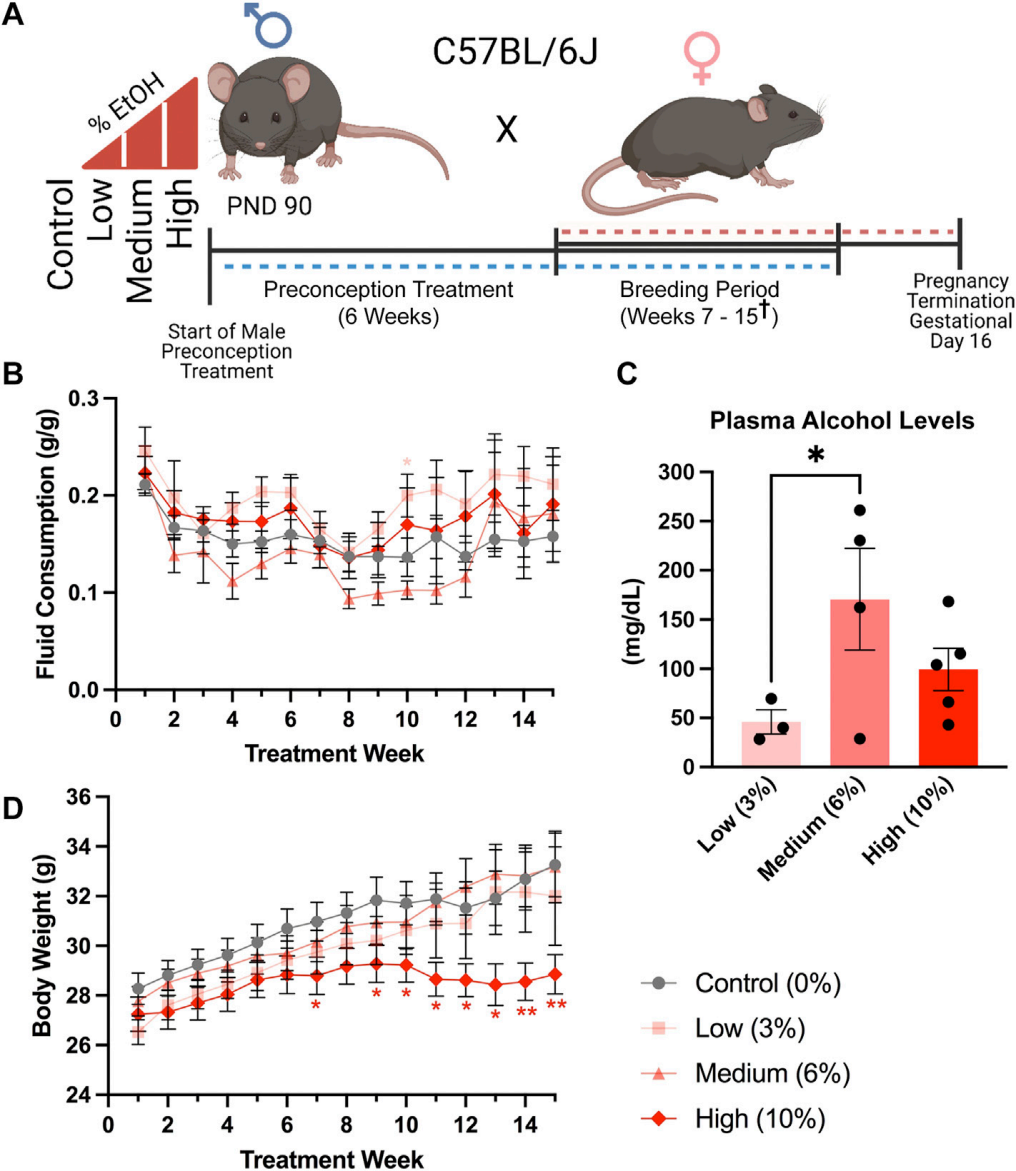
A limited access model to study concentration-dependent, ethanol-induced changes in paternal epigenetic programming. **(A)** Schematic outline of the experimental approach used to model the impacts of increasing ethanol concentrations on offspring fetoplacental growth. The preconception exposure period lasted 6 weeks, with exposures continuing during the breeding phase, which lasted from weeks seven and fifteen (^†^ one Control, two Medium- and two High-concentration litters were sired between weeks sixteen and eighteen). **(B)** Fluid consumption patterns compared between the different treatment groups (*n* = 17 Control, 13 Low, 12 Medium, 13 High). **(C)** Comparison of average plasma alcohol levels between treatment groups, measured after 3 weeks of exposure, at the end of the daily treatment window (*n* = 3 Low, 4 Medium, 5 High). **(D)** Comparison of sire body weight throughout the 10-week preconception exposure window (*n* = 17 Control, 13 Low, 12 Medium, 13 High). We used one-way **(C)** and two-way ANOVAs **(B,D)** to assay differences between treatment groups. Error bars represent the standard error of the mean, *p < 0.05, **p < 0.01.

Before initiating the EtOH and Control preconception treatments, we acclimated male mice to individual housing conditions for 1 week. After this acclimation period, we employed a modified version of the Drinking in the Dark model of voluntary alcohol consumption, initially described by Rhodes et al. (2005). Beginning 3 h into the dark phase, we exposed postnatal day 90 male mice to one of four preconception treatments by replacing the water bottle of their home cage with a bottle containing either: 0% (Control), 3% (Low-concentration), 6% (Medium-concentration), or 10% (High-concentration) w/v ethanol (catalog# E7023; Millipore-Sigma, St. Louis, MO, United States). We simultaneously exchanged the water bottles of Control and ethanol-exposed males to ensure identical handling and stressors. We exposed males to these treatments for 4 h every day and maintained the treatments for 6 weeks (preconception period, Figure 1A), which encompasses one complete spermatogenic cycle in mice (Adler, 1996). After the 6-week preconception exposure period, we began mating exposed males to naive dams but maintained the preconception treatments during this period. We bred exposed males to naive postnatal day 90 C57BL/6J females by first synchronizing the female reproductive cycle using the Whitten method (Whitten et al., 1968), then placing the female in the male’s home cage immediately after the male’s daily exposure window. 6 h later, we confirmed matings by the presence of a vaginal plug and returned females to their original cages. We rested treated males for 72 h, during which the males continued their exposures and then used them again in a subsequent mating. The breeding window lasted 1–12 weeks (7–18 weeks total exposure), depending on the time required to generate the requisite number of litters for each treatment group (Supplementary Table S1).

### Sire fluid consumption

We recorded treatment bottle and sire bodyweights weekly. We then quantified weekly fluid consumption by calculating the grams of fluid consumed divided by sire body weight (g/g). Subsequently, we determined the daily ethanol dose (g/kg) by multiplying the weekly fluid consumption (g/g) by the treatment group concentration (0.03, 0.06, or 0.10) and then divided this number by 7 days. Finally, for consistency with clinical studies (Leeman et al., 2010), we converted this number to grams per kilogram (g/kg).

### Plasma alcohol concentration

After 3 weeks of exposure, we isolated plasma from a subset of exposed males at the end of the 4-h exposure window. We then measured plasma alcohol concentrations using the AM1 Alcohol Analyser (Analox Technologies, Toronto, ON, Canada), according to the manufacturer’s protocol.

### Fetal dissection and tissue collection

We marked gestational day (GD) 0 by the presence of a copulation plug and recorded female body weight. We diagnosed pregnancies on gestational day ten by an increase in bodyweight of at least 1.8 g. After diagnosing pregnancy, we added additional nesting material (two nestlets) to the female’s home cage. We terminated dams on gestational day 16.5 using carbon dioxide asphyxiation followed by cervical dislocation. Subsequently, we dissected the female reproductive tract and recorded fetoplacental measures. We either fixed tissue samples in 10% neutral buffered formalin (catalog# 16004-128, VWR, Radnor, PA, United States) or snap-froze the tissues on dry ice and stored them at −80°C.

### Fetal sex determination

We isolated genomic DNA from the fetal tail using the HotSHOT method (Truett et al., 2000) and determined fetal sex using a PCR-based assay described previously (Thomas et al., 2021).

### Sire tissue collection

We terminated alcohol-exposed males using carbon dioxide asphyxiation and subsequent cervical dislocation. We dissected the testes, seminal vesicle, epididymal tract, liver, pancreas, and spleen, obtained tissue weights, and snap-froze on dry ice before long-term storage at −80°C.

### Placental RNA isolation and RT-qPCR gene expression

We isolated RNA from gestational day 16.5 placentae using the Qiagen RNeasy Plus Mini Kit (cat# 74136, Qiagen, Germantown MD, United States) and seeded approximately one υg of isolated RNA into a reverse transcription reaction using the High-Capacity cDNA Reverse Transcription Kit (catalog# 4368814, Thermo-Fisher, Waltham, MA, United States). Using published methods (Thomas et al., 2021), we determined the relative levels of candidate gene transcripts using the Dynamo Flash SYBR qPCR kit (catalog#F-415XL: Thermo-Fisher, Waltham, MA, United States). We describe the data normalization and handling procedures below; primer sequences are in Table 1.

**TABLE 1:** RT-qPCR primers.

Gene	Forward	Reverse
Ywhaz	TTG​ATC​CCC​AAT​GCT​TCG​C	CAG​CAA​CCT​CGG​CCA​AGT​AA
Pgk1	AGA​TGC​CAG​GAC​CTG​TAT​GCT​T	TGT​GCC​AGG​GTG​GTG​ACT​TTA
Slc22a18	TGA​TGT​CCA​GTG​TGC​TCC​AT	AGA​GTT​CGG​GTC​AAT​GGT​TG
Slc3a2	TGA​TGA​ATG​CAC​CCT​TGT​ACT​TG	GCT​CCC​CAG​TGA​AAG​TGG​A
Slc38a2	ACT​CAT​ACC​CCA​CCA​AGC​AG	CAC​AAT​CGC​ATT​GCT​CAG​AT
Slc38a4	TGA​TTG​GGA​TGT​TAG​TCT​GAG​G	GGC​CTG​GGT​TAA​AAT​GTG​TG
Slc2a3	GAT​CGG​CTC​TTT​CCA​GTT​TG	CAA​TCA​TGC​CAC​CAA​CAG​AG
Tpbpa	TGA​AGA​GCT​GAA​CCA​CTG​GA	CTT​GCA​GTT​CAG​CAT​CCA​AC
Cdkn1c	AAC​GTC​TGA​GAT​GAG​TTA​GTT​TAG​AGG	AAG​CCC​AGA​GTT​CTT​CCA​TCG​T
H19	TGA​TGG​AGA​GGA​CAG​AAG​GGC	CTT​GAT​TCA​GAA​CGA​GAC​GGA​CT
mPeg3	TTCTCCTTGGTCTCSCGGGC	AAG​GCT​CTG​GTT​GAC​AGT​CGT​G
Ascl2	TGC​CGC​ACC​AGA​ACT​CGT​AG	GCC​TCG​GTT​GCT​CCA​GAT​C
Mt-ND5	CCT​GGC​AGA​CGA​ACA​AGA​CAT	GGC​GAG​GCT​TCC​GAT​TAC​TA
Mt-Cytb	CAA​TCG​TTC​ACC​TCC​TCT​TCC​T	GAG​CGT​AGA​ATG​GCG​TAT​GC
Mt-Co1	CAA​TAG​TAG​AAG​CAG​GAG​CAG​GAA	GTT​TAG​GTT​GCG​GTC​TGT​TAG​TAG​T
Mt-Nd1	ATT​CTA​ATC​GCC​ATA​GCC​TTC​CT	TGG​GTG​TGG​TAT​TGG​TAG​GGG
Atp5e	GAC​AGG​CTG​GAC​TCA​GCT​AC	CCC​GAA​GTC​TTC​TCA​GCG​TT
Atp5mf	CGG​ACA​CCA​GGA​CTT​CAA​GAT	GGG​ACC​CCT​CTT​CAG​TGG​A
Atp5l	TAC​TCG​AAG​CCT​CGA​TTG​GC	AGG​GAT​TTC​AGC​AGG​GGT​TG

### Placental histological analysis

To increase tissue contrast, we treated tissue samples with phosphotungstic acid (Lesciotto et al., 2020) and processed them for MicroComputed tomography (microCT) imaging using methods described previously (Thomas et al., 2021). We embedded treated tissues in a 50/50 mixture of polyester and paraffin wax to prevent tissue desiccation during scanning. We imaged treated samples using a SCANCO vivaCT 40 (SCANCO Medical AG Brüttisellen, Switzerland) using a 55 kVp voltage x-ray tube and 29 uA exposure. The resulting microCT image voxel size was 0.0105 mm^3^, with 95.2381 pixels/mm resolution. Finally, we used the open-source medical image analysis software Horos (Version 3.3.6; Nibble Co. LLC, Annapolis, Maryland, United States; https://horosproject.org/) to quantify layer-specific volumes, as described previously (Clercq et al., 2019; Thomas et al., 2021).

After tissue scanning, we removed the embedding wax, washed samples in PBS, and processed them for histological analysis using a TP1020 Automatic Benchtop Tissue Processor (Leica, Deer Park, IL, United States). After processing, we paraffin-embedded and sectioned the samples using an RM2255 Rotary Microtome (Leica, Deer Park, IL, United States) and stained slides using a Periodic Acid Schiff (PAS) Stain Kit (Mucin Stain) (catalog #ab150680, Abcam, Boston, MA, United States) following the manufacturer’s provided protocol. We imaged samples using the VS120 Virtual Slide Microscope (Olympus, Waltham, MA, United States) and analyzed the images with the included desktop software, OlyVIA (Version 2.8, Olympus Soft Imaging Solutions GmbH, Muenster, Germany). Next, we quantified vascular space using methods described by Neres et al. (2008). Briefly, using the OlyVia software package, we selected microscopic fields from the labyrinth, one central and one peripheral, each with a magnification of ×20 and a resolution of 19029 × 9029). Using Photoshop (Version 21.0.1, Adobe, San Jose, CA, United States), we standardized the levels, parameters, and saturation of each image and converted the color schemes to black and white, with the cyan color of the blood cells adjusted to black and surrounding tissue showing in gray. Using ImageJ (Version 1.53f51, Wayne Rasband and contributors, National Institutes of Health, United States), we set a threshold to cover the entire tissue area (the gray pixels), excluding open vascular spaces and the area of any blood cells remaining in the tissue. We then used ImageJ to calculate the percent area that was grey, which we subtracted from 100 to derive the percent area of negative (vascular) space (Neres et al., 2008).

### Data handling and statistical analysis

All data generated during this study was subjected to a detailed data management plan that prioritizes safe and efficient data handling and allows long-term storage, retrieval, and preservation. We initially collected physiological measures by hand and then transcribed these data into Google Sheets or Microsoft Excel, where we collated and then transferred them into the statistical analysis program GraphPad Prism 8 (RRID:SCR_002798; GraphPad Software, Inc., La Jolla, CA, United States). For all analyses, we set the statistical significance at alpha = 0.05, used the ROUT test (*Q* = 1%) to identify outliers, and then verified the normality of the datasets using the Shapiro–Wilk test. If data passed normality (alpha = 0.05), we then employed either an ANOVA or an unpaired (two-tailed) *t*-test. If the data failed the test for normality, we then used an unpaired, non-parametric Mann–Whitney test. For measures of fetoplacental growth, we determined the male and female average for each litter and used this value as the individual statistical unit. We calculated placental efficiency (Hayward et al., 2016) by dividing fetal weight by placental weight, then deriving the male and female average for each litter. For offspring organ weights and the analysis of placental histology, we randomly selected male and female offspring from each litter and used measures of these samples as the statistical unit. Graphical depictions of data represent the mean and standard error of the mean. We provide detailed descriptions of each statistical test in Table 2.

**TABLE 2:** Statistical analyses associated with each figure.

Graph	Statistical test	Sample size	Outliers	
Figure 1: Experimental design for paternal alcohol exposure				
B. Sire fluid consumption by treatment week D. Sire body weight by treatment week	Two-way ordinary ANOVA, multiple comparisons using uncorrected Fisher’s LSD, only comparisons to Control were performed	*n* = 17 Control, 13 low, 12 Medium, 13 High	0	
C. Sire plasma alcohol levels	One-way ordinary ANOVA, multiple comparisons using uncorrected Fisher’s LSD	*n* = 3 Low, 4 Medium, 5 High	0	
Figure 2: Sire physiological data				
A-F. Testes, seminal vesicle, epididymal tract, liver, pancreas, spleen normalized to body weight G. Litter size	We inserted organ weights into Excel and combined the weights for paired (eg., left right testis) tissues, then divided by total body weight One-way ordinary ANOVA, multiple comparisons using uncorrected Fisher’s LSD	A-C. *n* = 22 Control, 12-25 Low, 15 Medium, 18 High	0	
		D-F. *n* = 22 Control, 9 Low, 10-15 Medium, 17 High				
		G. *n* = 22 Control, 25 Low, 15 Medium, 18 High				
Figure 3. Fetal and placental physiological data				
A-G. Gestational sac weight, crown-rump length, fetal weight, placental weight, placental diameter, placental efficiency, brain to body weight	Two-way ordinary ANOVA, multiple comparisons using uncorrected Fisher’s LSD, only comparisons to Control were performed	A-D/F. Males: *n* = 20 Control, 24 Low, 15 Medium, 18 High;	A-C & E-G. 0. D. Male: 2 Control, 1 Low, 1 High	
		Females: *n* = 21 Control, 25 Low, 15 Medium, 18 High [*n* = litter average]				
		E. *n* = 15 Control, 13 Low, 11 Medium, 17 High [*n* = litter average]				
		G. Males: *n* = 28 Control, 33 Low, 15 Medium, 16 High;				
		Females: *n* = 29 Control, 51 Low, 19 Medium, 17 High [*n* = fetus]				
Figure 4. Drinking types within the 10% (High) treatment group				
A. Fluid consumption determined drinking types within High group	Split population along average consumption of 0.157 g/g	*n* = 22 Control, 9 Moderate, 9 Heavy	0	
	One-way ANOVA, multiple comparisons using uncorrected Fisher’s LSD					
B. Placental weights for male and female fetuses by sire drinker type	Two-way ANOVA, multiple comparisons using uncorrected Fisher’s LSD	*n* = 22 Control, 9 moderate,9 heavy [*n* = litter average]	Male: 2 Control, 2 Heavy; Female: 1 Control, 2 Moderate	
C. Male placental weight vs. Average fluid consumption	Simple linear regression and two-tailed Pearson correlation	*n* = 18 [*n* = litter average]	0	
Figure 5. Nonlinear models examining the relationship between paternal dose and placental and fetal phenotypes				
A. Male and B. female log transformed relative average placental weight compared to sire daily ethanol dose	We normalized placental weights to the Control average, then log-transformed the average relative placental weights for each litter (dependent variable) and graphed these against the paternal dose of EtOH.	*n* = 29 [*n* = averaged individuals at each absolute daily dose]	0	
	Non-linear regression using fourth order polynomial model with least squares regression; Diagnostics: R squared and Runs test					
C. Male and female average relative placental weight and D. crown-rump length compared between upper and lower thresholds (inflection points)	Two-way ordinary ANOVA, multiple comparisons using uncorrected Fisher’s LSD	*n* = 10 lower (< 1.0) and 5 upper (> 2.4) [*n* = averaged individuals at each absolute daily dose]	0	
E. Male and F. female average relative crown-rump length compared to sire daily ethanol dose	Non-linear regression using fourth order polynomial model with least squares regression; Diagnostics: R squared and Runs test	*n* = 29 [*n* = averaged individuals at each absolute daily dose]	0	
Figure 6. Placental histological analysis				
B-E, H-I. Chorion, decidua, junctional zone, labyrinth, junctional zone to decidua, and labyrinth to junctional zone	Two-way ANOVA, multiple comparisons using uncorrected Fisher’s LSD	Males: *n* = 21 Control, 43 Low, 9 Medium, 11 High;	B, D-E, & I. 0 C. Male: 1 Low; Female: 1 Control H. Male: 1 Low	
		Females: *n* = 18 Control, 42 Low, 9 Medium, 9 High				
F/G. Male Dose Response Decidua and Labyrinth	Simple linear regression and two-tailed Pearson correlation	*n* = 13 [*n* = averaged individuals at each absolute daily dose]	0	
Figure 7. Placenta-Heart Analysis				
J. Male and female central and peripheral labyrinth blood spaces	Three-way (sex, location, treatment) ordinary ANOVA, multiple comparisons using uncorrected Fisher’s LSD	Males: 8 Control, 8 Low, 6 High	0	
		Females: 8 Control, 8 Low, 7 High				
K. Heart to body weight	Two-way ordinary ANOVA, multiple comparisons using uncorrected Fisher’s LSD, only comparisons to Control were performed	Males: *n* = 13 Control, 16 Low	0	
		Females: *n* = 11 Control, 25 Low				
Figure 8. Placental gene expression analysis				
Gene	Statistical Test	Experimental N		
		Outliers		
		Control	Low	High
Ascl2	M: Welch; F: ANOVA	M7; F8	M7; F7	M8; F8
		0	0	M3; F0
Cdkn1c	M: Welch; F: ANOVA	M 8; F8	M7; F8	M8; F7
		0	0	0
Slc22a18	M & F: Welch	M8; F8	M7; F7	M8; F8
		M0; F1	M0; F0	M0; F1
Slc3a2	M: ANOVA; F: Kruskal—Wallis	M7; F7	M8; F7	M8; F8
		M1; F0	M0; F0	M3; F0
Tpbpa	M: Welch; F: ANOVA	M7; F6	M7; F7	M8; F8
		M1; F0	M0; F1	M0; F0
Mt—Cytb	M: ANOVA; F: Kruskal—Wallis	M7; F8	M7; F7	M8; F8
		M0; F1	M2; F2	0
H19	M: ANOVA; F: Welch	M7; F8	M7; F7	M7; F8
		0	0	0
mPeg3	F: Kruskal—Wallis	M7; F8	M7; F7	M7; F8
		0	0	0
Mt—Nd5	M & F ANOVA	M8; F7	M8; F5	M8; F8
		0	0	0

### RT-qPCR analysis of placental gene expression

We imported the replicate cycle threshold (Ct) values for each transcript into Excel and normalized the expression to the geometric mean of two reference genes. These included transcripts encoding *Phosphoglycerate kinase 1* (*Pgk1*) and *3-monooxygenase/tryptophan 5-monooxygenase activation protein zeta* (*Ywhaz*). We used the −∆∆CT Method (Schmittgen and Livak, 2008) to calculate the relative fold change for each replicate. After combining datasets in excel, we input the values into the statistical analysis program GraphPad Prism eight and identified outliers using the ROUT outlier test (*Q* = 1%). Next, we verified all datasets for normality using the Shapiro-Wilk test. If data passed normality (alpha = 0.05), we employed a One-Way ANOVA or, if the standard deviations were significantly different (Browne-Forsythe), a Welch ANOVA. If normality failed, we performed a non-parametric Kruskal-Wallis test.

## Results

### A limited voluntary access model to examine concentration-dependent effects of chronic paternal alcohol exposure

In our previous studies utilizing a prolonged version of the Drinking in the Dark model, male C57BL/6J mice consumed an average of 0.12 g/g/week (Chang et al., 2019a). These published studies utilized a 10% (w/v) mixture of alcohol and 0.001% Sweet’N Low^®^ to increase palatability. In the current study, we eliminated the Sweet n Low and administered 3, 6, and 10% (w/v) ethanol (EtOH) solutions. We exposed Control males to water alone and ensured identical handling by concurrently switching between two identical water bottles. In addition, we strictly enforced consumption rates and eliminated males that dropped below a weekly fluid consumption of 0.08 g/g/week, one standard deviation below the previously identified average, for more than three consecutive weeks. In total, we eliminated 0 Low-concentration (3%), 1 Medium-concentration (6%), and 10 High-concentration (10%) males (Supplementary Table S2).

We exposed males to the preconception treatments for 6 weeks, encompassing one complete murine spermatogenic cycle (Adler, 1996) (Figure 1A). We did not observe any differences in fluid consumption between treatment groups during the preconception treatment period. However, at week ten, during the breeding phase (weeks seven to fifteen), males in the Low-concentration group consumed more fluid than the Control treatment (*p* = 0.0334), while Medium-concentration males consumed more than the High-concentration group (*p* = 0.0428), but not Controls (Figure 1B). After 3 weeks of exposure, we collected blood at the end of the 4-h exposure window and measured plasma alcohol levels. We observed an average of ∼50 mg/dl in the Low-concentration group, ∼150 mg/dl in the Medium-concentration group, and ∼100 mg/dl in the High-concentration group (Figure 1C). The plasma alcohol levels we observed in the Medium- and High-concentration groups were not significantly different and fell within the range previously identified using the Drinking in the Dark model (100–160 mg/dl) (Rhodes et al., 2005; Thiele et al., 2014). Interestingly, plasma alcohol concentrations in the Low-concentration group were significantly lower than those in the Medium-concentration males and below levels observed in previous studies using this same exposure model. We speculate that differences in the palatability of the different concentrations lead to changes in consumption patterns and resulting plasma alcohol concentrations. However, as stress is known to modify the paternally-inherited epigenome (Chan et al., 2018), we subjected males to minimal handling and only measured plasma alcohol concentrations once. During the breeding phase, weeks seven to fifteen, we observed a significant decline in the bodyweights of males within the High-concentration treatment group (Figure 1D). We did not observe differences in weekly body weights between any other treatment groups.

**FIGURE 2:**
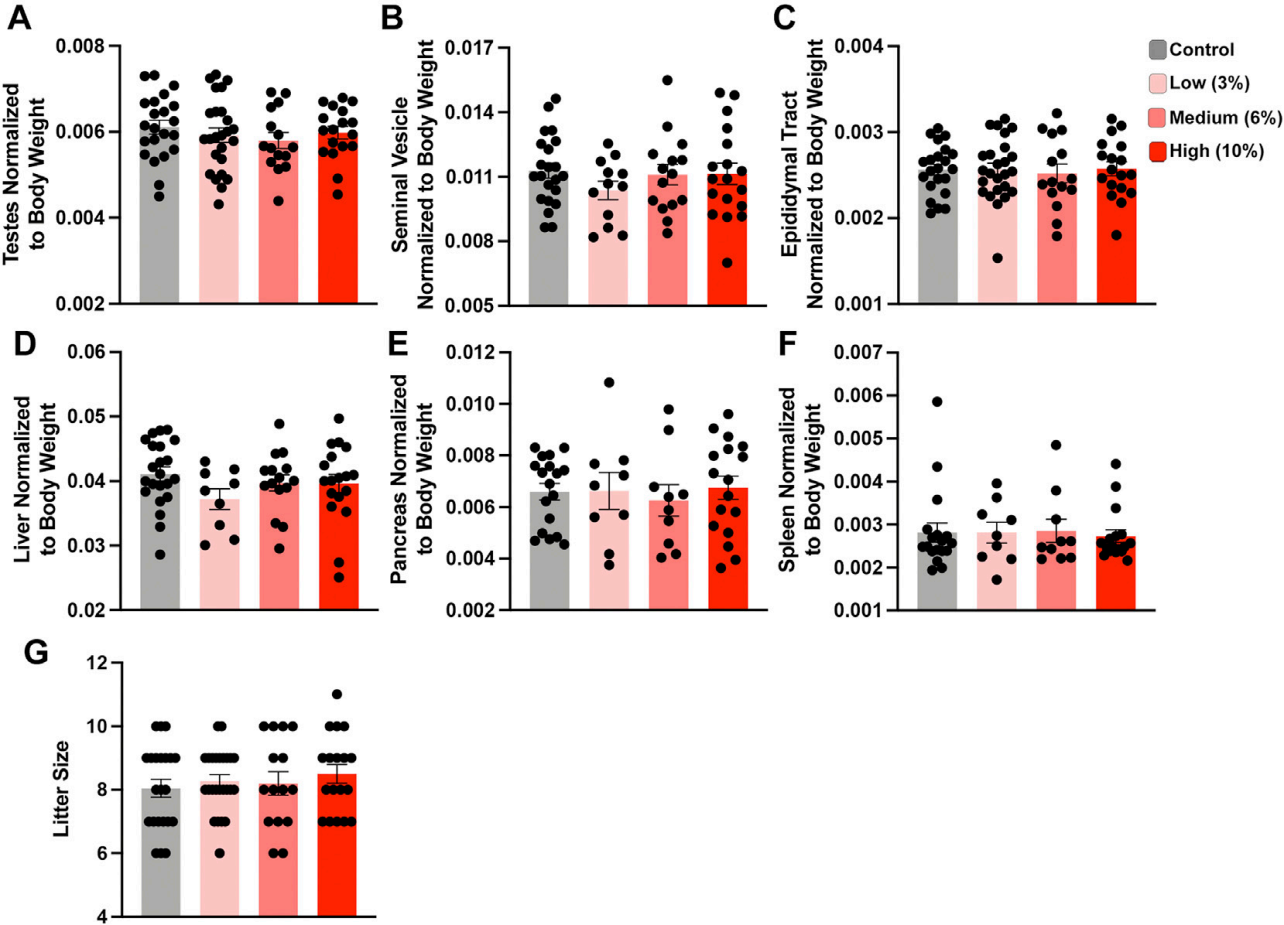
Chronic paternal ethanol exposures do not impact macro measures of male reproductive health or fertility. Paternal preconception alcohol exposures do not impact large-scale measures of male reproductive physiology, including normalized **(A)** testis, **(B)** seminal vesicle, or **(C)** epididymal track weights (*n* = 22 Control, 12-25 Low, 15 Medium, 18 High). Chronic male ethanol exposure does not impact normalized **(D)** liver, **(E)** pancreas or **(F)** spleen weights (*n* = 22 Control, 9 Low, 10-15 Medium, 17 High). **(G)** Chronic preconception paternal alcohol exposures do not influence offspring litter size (*n* = 22 Control, 25 Low, 15 Medium, 18 High litters). To assay changes between treatment groups, we used a one-way ANOVA. Error bars represent the standard error of the mean.

The average time to conception was ∼4 weeks (10 weeks of total exposure) for the Control and Low-concentration treatments and 5.2 and 5.8 weeks for the Medium- and High-concentration treatments (11.2 and 11.8 weeks of total EtOH exposure) (Supplementary Table S1). At week 15, we sacrificed the exposure cohort, with the exception of five males (1 Control, 2 Medium- and 2 High-concentration), which required up to three more weeks to sire litters (Supplementary Table S1). At sacrifice, we dissected Control and EtOH-exposed sires and examined physiologic measures of their general and reproductive health. We did not observe any differences in the normalized weights of the testes, seminal vesicles, or epididymides, nor any changes in the liver, pancreas, and spleen (Figures 2A–F). In addition, consistent with previous studies, none of the examined alcohol treatments impacted litter size (Figure 2G). Based on these data, we conclude that none of the examined EtOH treatments negatively impact large-scale measures of male reproductive physiology.

### Chronic low- and medium-concentration preconception ethanol exposures impact offspring fetoplacental growth

**FIGURE 3:**
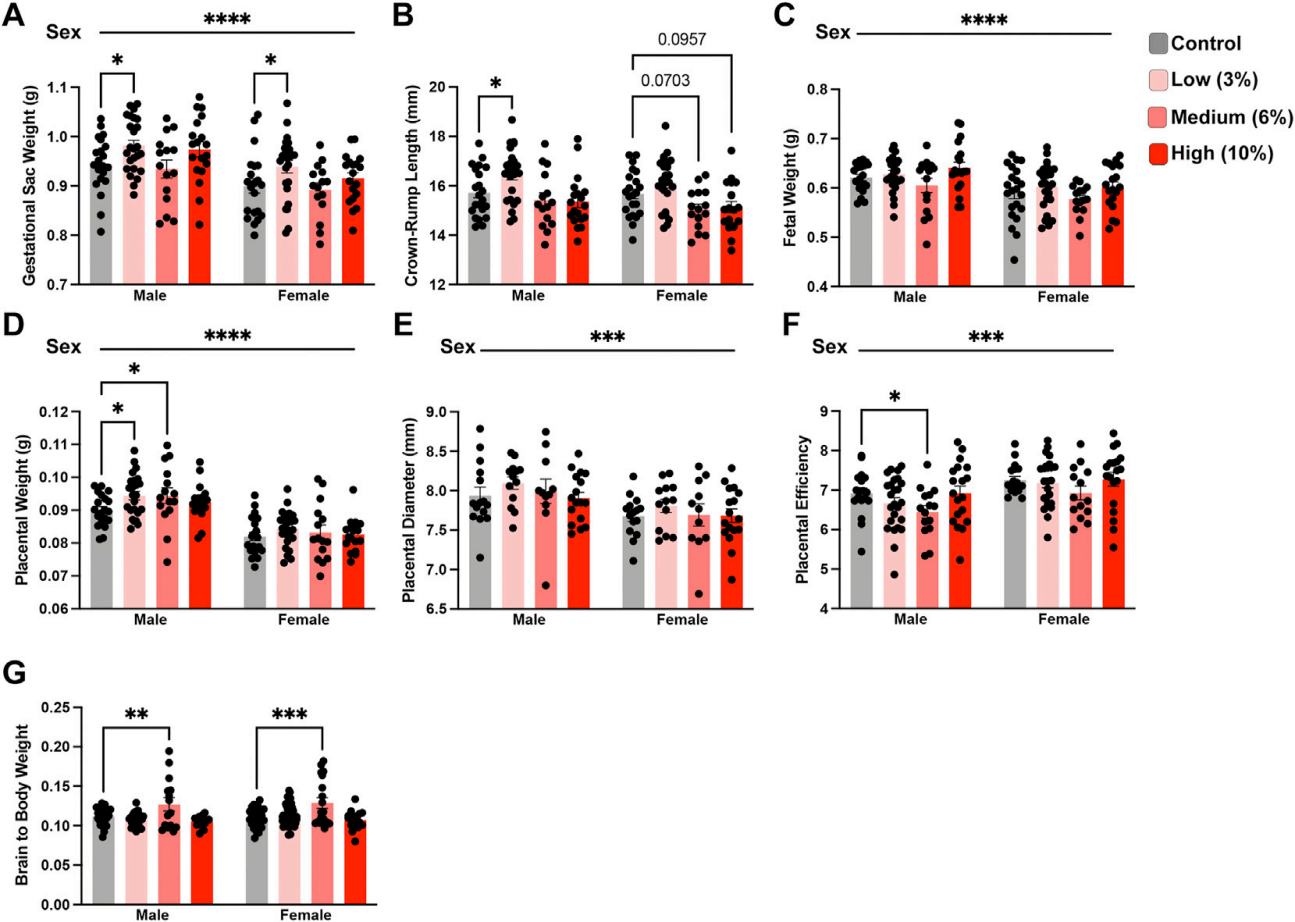
Paternal preconception alcohol exposure induces concentration-dependent changes in offspring fetoplacental growth. For all measures of fetoplacental growth, we used the male and female average for each litter as the individual statistical unit. Comparison of litter average **(A)** gestational sac weight, **(B)** crown-rump length, **(C)** fetal weight, **(D)** placental weight, **(E)** placental diameter, and **(F)** placental efficiency between offspring sired by males across treatment groups (A–D/F. *n* = litter average with males: 20 Control, 24 Low, 15 Medium, 18 High litters; females: 21 Control, 25 Low, 15 Medium, 18 High litters; **(E)** *n* = litter average with males and females: 15 Control, 13 Low, 11 Medium, 17 High litters). **(G)** Comparison of normalized brain weights in the offspring of alcohol-exposed males across treatment groups (*n* = fetus, randomly selected from each litter, males: 28 Control, 33 Low, 15 Medium, 16 High; females: *n* = 29 Control, 51 Low, 19 Medium, 17 High). We used either a two-way ANOVA to contrast the impacts of sex and preconception treatments or Brown-Forsythe and Welch’s one-way ANOVA. Sex differences are indicated above the figures, while treatment effects are demarcated directly above the bar graphs. Error bars represent the standard error of the mean, *p < 0.05, **p < 0.01, ***p < 0.001, ****p < 0.0001.

We next examined the intergenerational impact preconception paternal exposure to varying concentrations of EtOH has on fetoplacental growth by assessing offspring physiologic measures at gestational day 16.5 (GD16.5). This gestational phase represents a period where placental weights have reached their maximum, and fetal growth is exponential (Mu et al., 2008). Further, we have previously identified EtOH-induced placental overgrowth and fetal growth restriction at this stage (Chang et al., 2017; Bedi et al., 2019; Thomas et al., 2021). Using a one-way ANOVA, we did not find any differences in gestational day 16.5 dam weights normalized to litter size (Supplementary Figure S1A). Next, using a two-way ANOVA, we contrasted male and female physiologic measures of offspring growth across treatment groups. Unexpectedly, offspring derived from sires in the Low-concentration treatment group exhibited increased average gestational sac weights, crown-rump lengths, and placental weights (Figure 3A–F). However, the differences in crown-rump length and placental weights only appeared in the male offspring. In contrast, the offspring of Medium-concentration sires only displayed increased male placental weights, while we did not observe differences in any parameters measured in offspring derived from the High-concentration treatment group. Next, we wondered if an extended exposure duration could drive a progressive increase in litter average placental weights. To address this question, we combined the Low- and Medium-concentration datasets and compared measures before and after 11 weeks, the mean time to conception for both groups. However, this comparison did not reveal any significant differences in placental growth (Supplemental Figures 1B–C).

Recent studies examining periconceptional maternal exposures suggest that EtOH may impact brain weights in the developing offspring (Steane et al., 2021). Further, increased proportional brain to body weight is a reliable indicator of intrauterine growth restriction (IUGR) (Cortes-Araya et al., 2022). Therefore, we examined the ratio of brain to total bodyweight in the male and female offspring. These analyses revealed that both male and female offspring from sires within the Medium-concentration treatment group exhibited increased normalized brain weights (Figure 3G). However, we did not observe any differences in normalized brain weights in offspring derived from sires in the Low and High-concentration treatment groups. These observations collectively indicate that preconception paternal EtOH exposure impacts multiple aspects of fetoplacental growth and suggests that several measures may exhibit concentration-specific outcomes.

### High-concentration paternal alcohol exposures induce dose-dependent effects on placental growth

**FIGURE 4:**
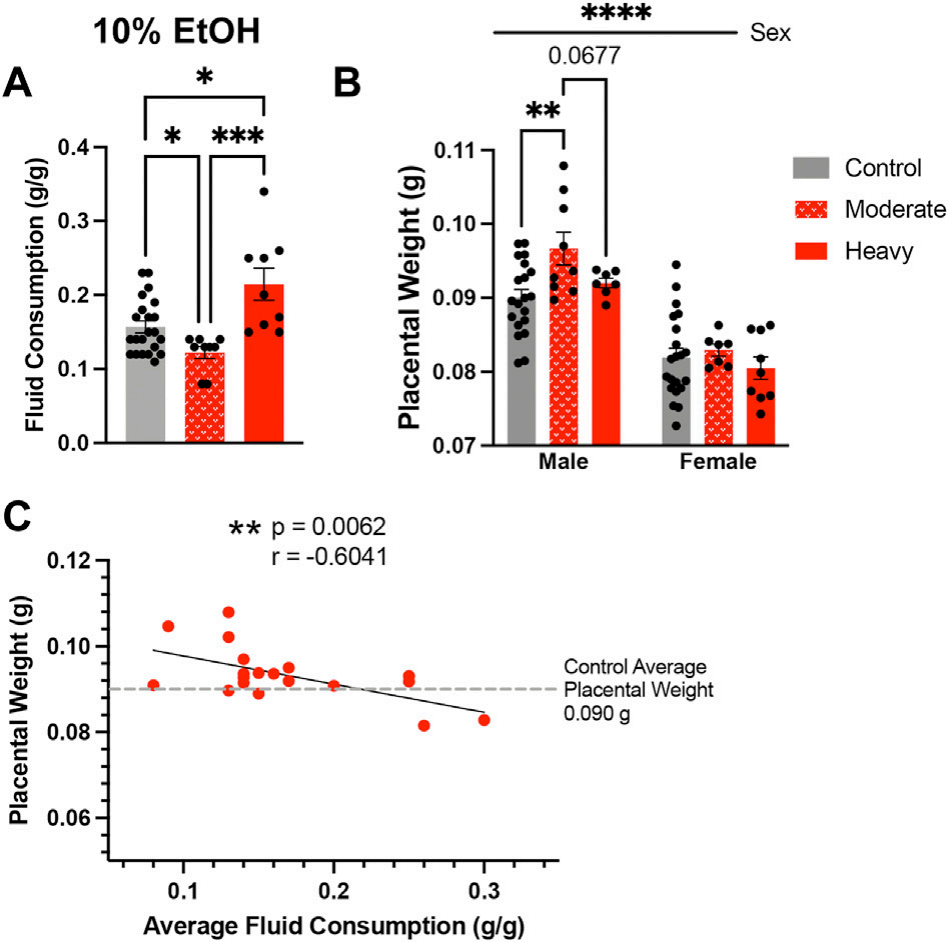
High-concentration sires display significant variation in alcohol consumption, which associates with differing effects on placental growth in the male offspring. **(A)** Comparison of average fluid consumption between the bottom and top consuming males within the High-concentration treatment group (One-way ANOVA, *n* = 22 Control, 9 Moderate, 9 Heavy). **(B)** Increased average placental weights in offspring sired by moderate but not heavy drinking males within the High-concentration treatment group (*n* = litter average with 22 Control, 9 Moderate, and 9 Heavy litters). We used a two-way ANOVA to compare average placental weights between the male and female offspring of Control, Moderate, and Heavy drinking sires. **(C)** Pearson correlation analysis contrasting male offspring litter average placental weights and average paternal fluid consumption across the High-concentration treatment group (*n* = 18 litters). Error bars represent the standard error of the mean, *p < 0.05, **p < 0.01, ***p < 0.001, ****p < 0.0001.

Given the consistent impact of preconception paternal 10% w/v EtOH exposures on placental weights observed previously (Chang et al., 2017; Chang et al., 2019a; Bedi et al., 2019; Thomas et al., 2021), we were surprised that we did not detect increased placental growth within offspring sired by males in the High-concentration treatment group. In analyzing sire fluid consumption data, we noted that, although similar to the Controls in the present study, the High-concentration treatment group drank more than males in our previous studies. Further, we also observed larger than anticipated within-group variation. The average fluid consumption level for Control males was 0.157 g/g. Therefore, we split the High-concentration sires into two populations, one below 0.157 g/g (moderate drinkers) and one above (heavy drinkers), and then compared fluid consumption between these males. This comparison revealed significant differences in fluid consumption between heavy and moderate drinkers (Figure 4A). Importantly, placental weights of male offspring sired by moderate drinkers were significantly higher than Controls, but those of heavy drinkers were not (Figure 4B). To further test the relationship between sire fluid consumption and offspring placental weights, we conducted a Pearson correlation analysis, which revealed increased paternal EtOH exposure correlated with a decline in both male (Figure 4C; *r* = −0.6041, *p*-value = 0.0062) and female (*r* = −0.5773, *p*-value = 0.0096; Supplementary Figure S2) placental weights. Significantly, these observations reveal that males within the intended treatment groups received different doses of alcohol and that paternal EtOH exposure may induce dose-dependent effects on placental growth.

### Chronic paternal ethanol exposures induce dose-dependent, hormetic effects on offspring fetoplacental growth

**FIGURE 5:**
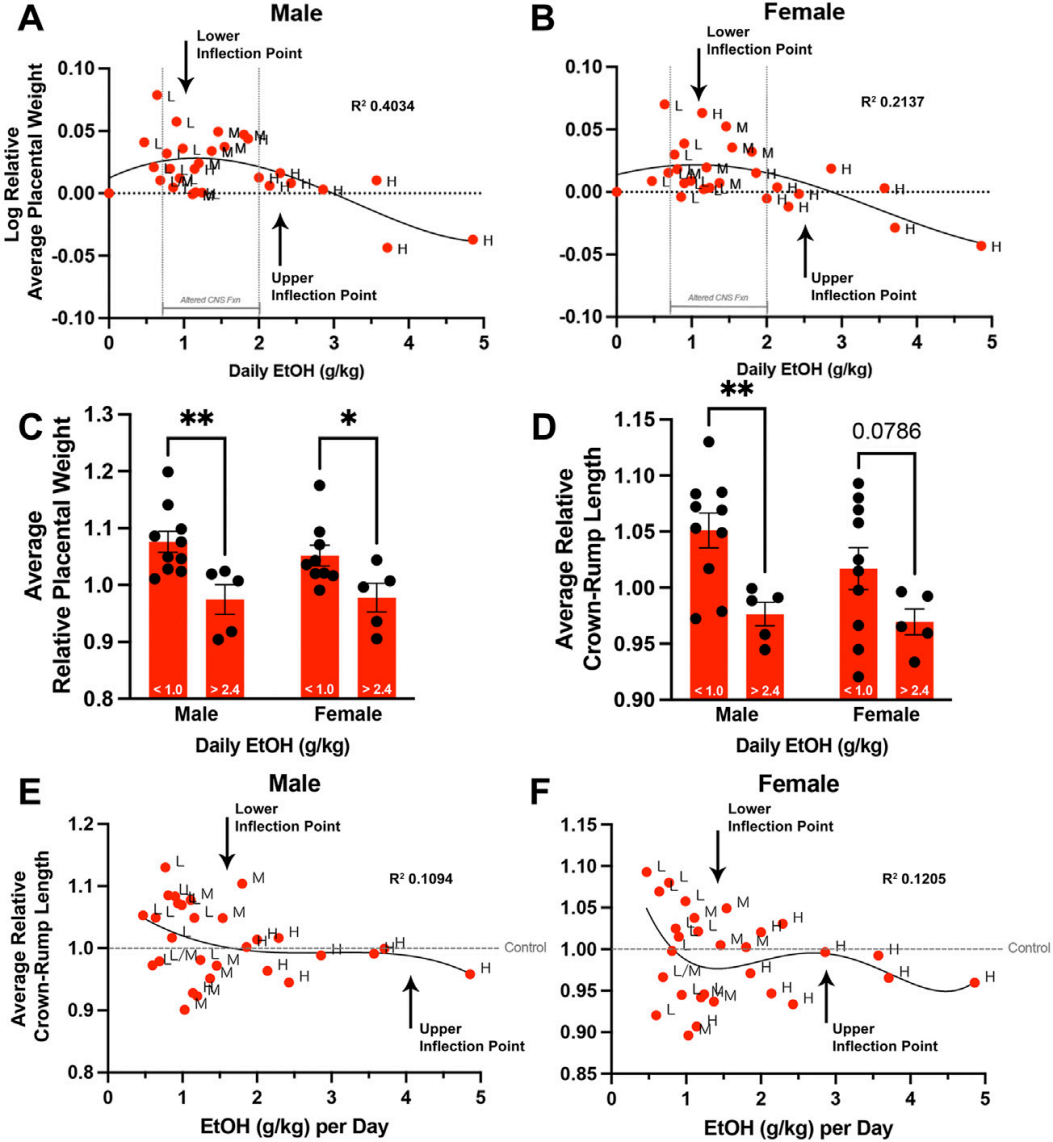
Chronic preconception paternal ethanol exposures induce biphasic, dose-dependent effects on offspring fetoplacental growth. We used nonlinear regression to compare the sire’s daily ethanol dose to the log-transformed, average relative placental weights of **(A)** male and **(B)** female offspring. (*n* = 29 averages across each absolute daily dose). Comparison of male and female **(C)** average relative placental weight and **(D)** average relative crown-rump lengths between offspring sired by males below (< 1.0 g/kg) and above (> 2.4 g/kg) the identified inflection points (two-way ANOVA with *n* = 10 lower-dose and 5 upper-dose). Nonlinear regression comparing sire daily ethanol dose to **(E)** male and **(F)** female average relative crown-rump lengths (*n* = 29 averages across each absolute daily dose). We used a fourth-order polynomial model with least squares regression to identify inflection points. We eliminated outliers at *Q* = 1% and verified model fit using R squared analyses combined with Runs testing. Error bars represent the standard error of the mean, *p < 0.05, **p < 0.01.

In addition to the variation in weekly fluid consumption observed across all treatment groups, we also observed significant differences in the within-group standard deviations of male and female placental weights (*p* < 0.05). This increased variation suggested a more complex interplay between paternal EtOH exposure and placental growth. As we could correlate increased paternal EtOH consumption with decreasing placental weights in the High-concentration group, we examined this correlation across all EtOH treatment groups. To test this, we first calculated the absolute daily dose of alcohol consumed in grams EtOH per kilogram body weight (see Materials and Methods). However, when examining the offspring of all three original treatment groups, we did not observe a linear relationship between paternal EtOH dose and placental weight (R^*2*^ < 0.05, Runs test *p* < 0.001). Numerous studies indicate that EtOH exhibits a nonlinear, J-shaped effect on many physiologic systems, with low doses exerting distinct toxic effects, hazard ratios, and neurological outcomes from higher doses (Kalev-Zylinska and During, 2007; Velazquez-Marrero et al., 2011; Fernández-Solà, 2015; Lundgaard et al., 2018; Sureshchandra et al., 2019). Based on these previous studies, we log-transformed the relative average placental weights for each litter (dependent variable) and graphed these against the paternal dose of EtOH. These analyses identified a sine-like relationship, or inverse J-curve, where lower levels of paternal EtOH consumption are associated with increased normalized placental weights. In contrast, relative placental weights decline at higher doses, eventually dropping below the Control group (male *p* = 0.0021, female *p* = 0.0317; Figures 5A,B; Supplementary Table S3). Using a fourth-order polynomial model, we identified a plateau or upper threshold at ∼1 g/kg daily dose and an inflection point at ∼2.4 g/kg per day, past which increasing paternal alcohol doses induced a decline in placental weights (Figures 5A,B). We then separated and compared fetoplacental measures below and above these identified points. These comparisons identified threshold-dependent differences in normalized litter average placental weights for male and female offspring (Figure 5C). Fetal crown-rump lengths were significantly different in males and showed a similar trend in female offspring (Figure 5D). We did not observe dose-dependent differences in litter average relative fetal weights for either sex (data not shown). Finally, we developed a nonlinear regression model to compare relative crown-rump length and sire ethanol dose for male (Figure 5E) and female offspring (Figure 5F). These analyses revealed that increases in fetal crown-rump length primarily correlate with increasing paternal EtOH exposures between doses ∼0.8–1.3 g/kg/day, past which we do not observe any impacts. Our studies reveal that paternal EtOH exposure induces nonlinear, dose-dependent changes to offspring placental growth and that low-level paternal exposures associate with male-biased increases in crown-rump length.

### Chronic paternal alcohol exposures exert dose-dependent changes on the histological organization of the placenta

**FIGURE 6:**
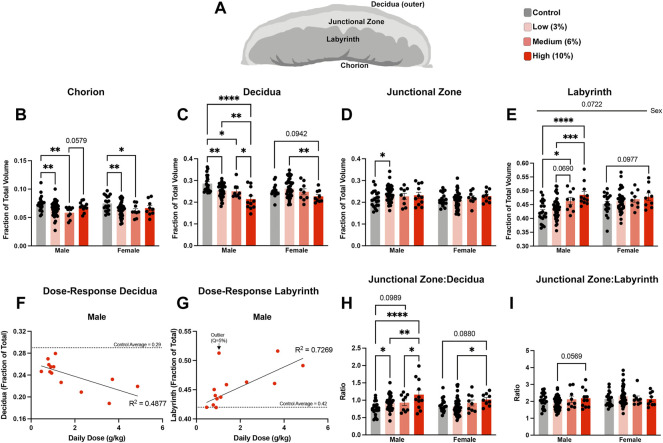
Chronic paternal alcohol exposures induce dose- and sex-specific changes in the histological organization of the placenta. **(A)** Schematic diagram depicting the layers of the murine placenta. Using microCT, we conducted a volumetric analysis of each placental layer and used a two-way ANOVA to compare measures between male and female offspring across treatment groups. Volumes for the **(B)** chorion, **(C)** decidua, **(D)** junctional zone, and **(E)** labyrinth are expressed as a ratio of the total placental volume (*n* = fetus, randomly selected from each litter, males: 21 Control, 43 low, 9 Medium, 11 High; females: *n* = 18 Control, 42 Low, 9 Medium, 9 High). Pearson correlation analysis contrasting proportional volume of the **(F)** decidua and **(G)** labyrinth with sire daily ethanol dose (*n* = 13 averaged individuals at each absolute daily dose). Ratios comparing the proportional volumes of the **(H)** junctional zone to decidua and **(I)** labyrinth to junctional zone between male and female offspring across treatment groups. Error bars represent the standard error of the mean, *p < 0.05, **p < 0.01, ***p < 0.001, ****p < 0.0001.

Using an outbred mouse model, we previously observed that preconception male EtOH exposure induces female-specific changes in the histological organization of the placenta (Thomas et al., 2021). To determine if we could identify concentration-specific changes in placental histology, we used microCT imaging to quantify the different placental layers (Clercq et al., 2019) (Figure 6A). Using phosphotungstic acid to enhance tissue contrast, we determined the proportional volumes of the chorion, labyrinth, junctional zone, and maternal decidua in placentae derived from the offspring of Control and EtOH-exposed sires. These experiments revealed that both the male and female offspring of Low- and Medium-concentration sires exhibited reductions in the chorion, while placentae derived from sires in the High-concentration treatment group did not (Figure 6B). Interestingly, we identified progressive, concentration-dependent reductions in the maternal decidua, which were accompanied by increases in the labyrinth layer, but only in the male offspring of EtOH-exposed sires (Figures 6C–E). For male placentae, Pearson correlation analysis confirmed linear, dose-dependent decreases in the volume of the decidua (R^*2*^ = 0.4877, *p*-value 0.0079), offset by dose-dependent increases in the volume of the labyrinth layer (R^*2*^ = 0.7269, *p*-value 0.0183) (Figures 6F,G; Supplementary Table S3). We did not observe any significant dose-response correlations in the proportional volumes of female placentae (decidua *p*-value 0.1028, labyrinth *p*-value 0.2110; Supplementary Table S3). Additionally, the male offspring of Low-concentration sires exhibited a modest increase in the junctional zone (Figure 6D). When we compared proportional ratios of each layer, we identified differences in the ratio of the junctional zone and decidua but not the proportional ratio of the junctional zone and labyrinth (Figures 6H,I), suggesting the endocrine component of the placenta may remain constant while the region responsible for maternofetal exchange expands with increasing paternal EtOH exposures.

**FIGURE 7:**
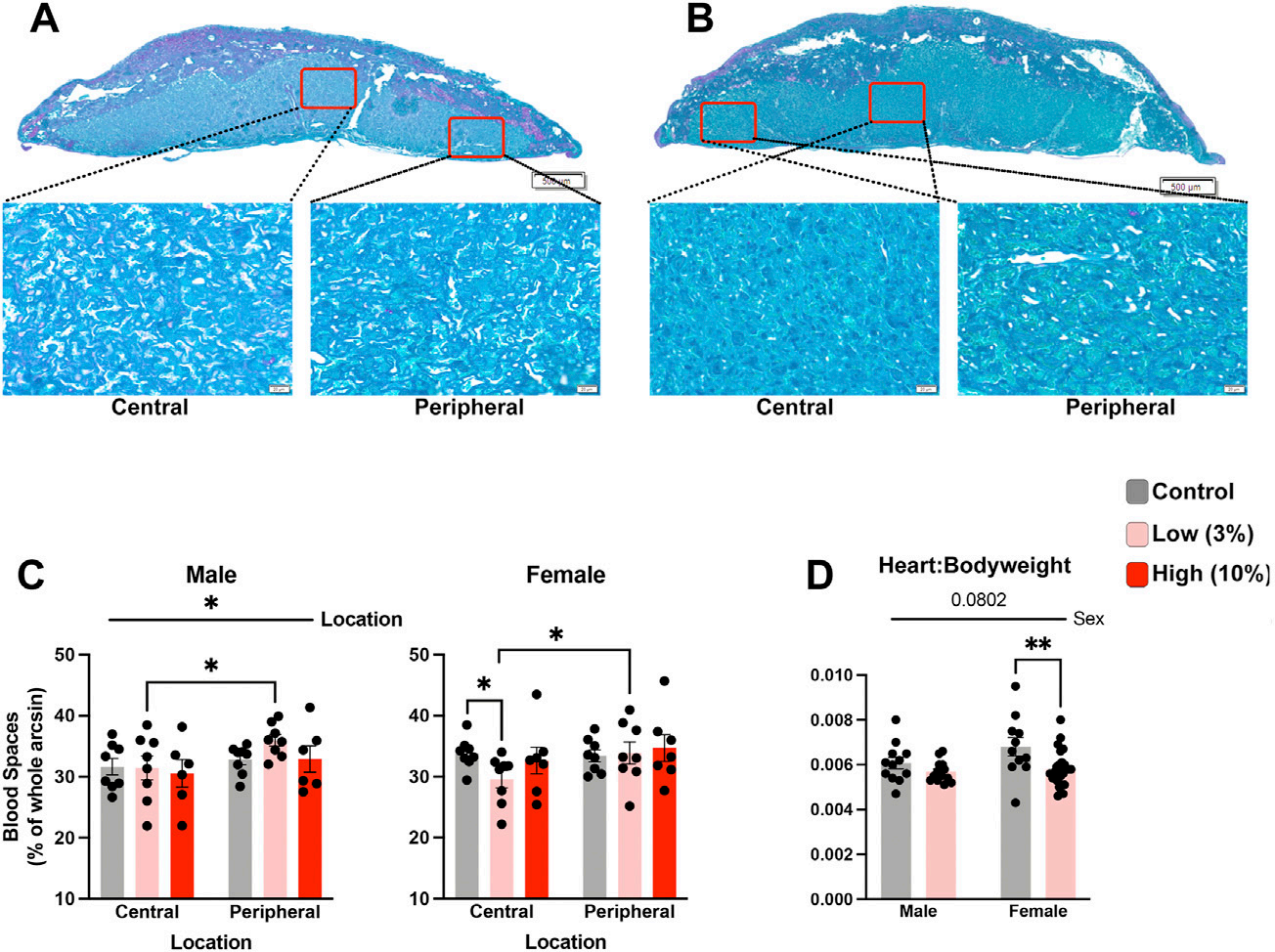
Low-concentration paternal alcohol exposures alter placental vascular space. Histological sections comparing central and peripheral vascular spaces between female placentae derived from the offspring of **(A)** Control and **(B)** Low-concentration sires. **(C)** Comparison of placental vascular space between male and female offspring of Control, Low-, and High-concentration sires (*n* = fetus, randomly selected from each litter, males: 8 Control, 8 low, 6 High; females: *n* = 8 Control, 8 Low, 7 High). **(D)** Comparison of relative heart weights between the male and female offspring of Control and Low-concentration sires (*n* = fetus, randomly selected from each litter, males: 13 Control, 16 low; females: *n* = 11 Control, 25 Low). We used a two-way ANOVA to contrast differences between sex and the preconception treatment groups. Error bars represent the standard error of the mean, *p < 0.05, **p < 0 0.01.

During our histological analysis, we noticed that the labyrinth zone in placentae of offspring sired by Low-concentration males appeared to have increased density compared to placentae derived from the offspring of Control males. For female offspring of EtOH-exposed sires, morphometric analysis of standard histological sections (Neres et al., 2008) revealed a reduction in placental vascular spaces within the central regions of the labyrinth compared to Controls but not in peripheral regions (Figures 7A–C). We did not observe changes in vascular space within placentae from male or female offspring derived from High-concentration sires. In contrast, although the density of the labyrinth region of Low-concentration male placentae was not statistically different from the Controls, the peripheral labyrinth of the Low-concentration placentae contained proportionally more vascular space than the central regions (Figure 7C). We did not observe proportional differences in central vs. peripheral vascular space in the Controls. These data indicate that while we do not observe alterations in the size of the labyrinth layer in female offspring, we do observe indications of altered vascular structure.

Finally, emerging research suggests a shared developmental axis between the placenta and heart (Woods et al., 2018), and a recent study examining low-level maternal alcohol exposures identified heart defects in the offspring (Nguyen et al., 2014). Therefore, we used a two-way ANOVA to compare normalized heart weights between the Control and Low-concentration treatment groups. This analysis revealed decreased normalized heart weights in the female offspring but not the males (Figure 7D). From these observations, we conclude that paternal, Low-concentration alcohol exposures exert sex-specific impacts on placental development, which correlates with sexually dimorphic reductions in offspring normalized heart weights.

#### Alterations in mitochondrial-encoded and imprinted gene expression accompany paternally programmed changes in placental histology

**FIGURE 8:**
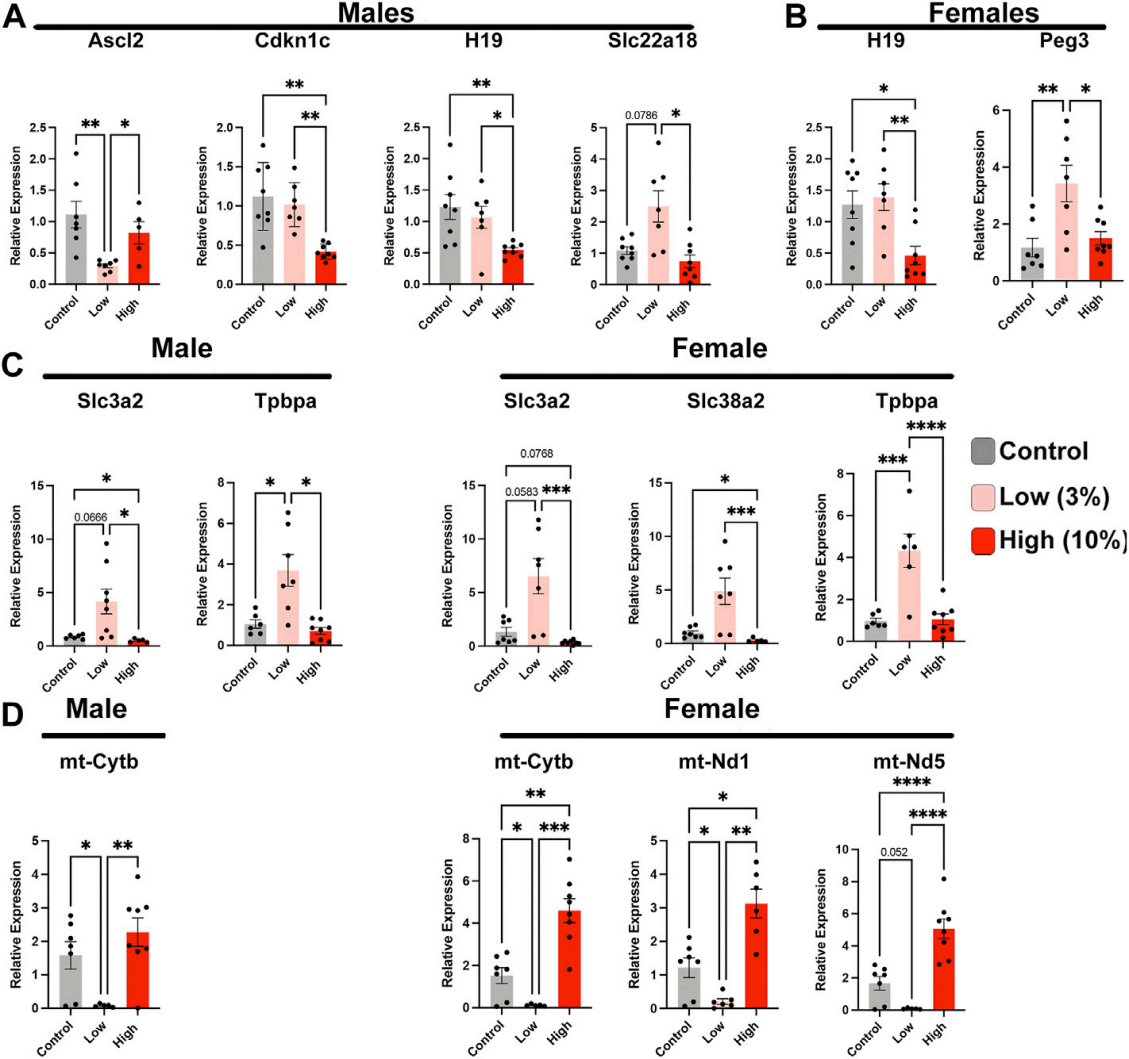
Paternal alcohol exposures induce alterations in placental gene expression. Analysis of imprinted gene expression in the placentae of **(A)** male and **(B)** female offspring of Control and EtOH-exposed sires in the Low- and High-concentration treatment groups. **(C)** Expression analysis of critical placental nutrient transporters in male and female offspring sired by Control, Low-, and High-concentration ethanol exposed males. **(D)** Comparison of mitochondrial-encoded transcripts in placentae derived from the male and female offspring of sires exposed to the Control, Low-, and High-concentration ethanol treatments. We analyzed gene expression using RT-qPCR. Gene expression was normalized to transcripts encoding *Pgk1* and *Ywhaz*; (*n* = 8). For analysis, we used a one-way ANOVA or a Welch ANOVA. If data were not normally distributed, we used a non-parametric Kruskal-Wallis test. Error bars represent the standard error of the mean, *p < 0.05, **p < 0.01, ***p < 0.001, ****p < 0.0001.

Epigenetic mechanisms of gene regulation, particularly imprinted genes, play a central role in directing the histological organization and function of the placenta (Piedrahita, 2011; Tunster et al., 2016). Therefore, we assayed the expression of a select cohort of imprinted genes with known roles in organizing placental histoarchitecture. In the male offspring of Low-concentration sires, we identified decreased expression of achaete-scute family BHLH transcription factor 2 (*Ascl2*) and increased expression of solute carrier family 22 member 18 (*Slc22a18*), both maternally expressed genes located in the *Kcnq1ot1* imprinted domain (Golding et al., 2011) (Figure 8A). In contrast, in placentae derived from the male offspring of high-concentration sires, we observed upregulation of cyclin-dependent kinase inhibitor 1C (*Cdkn1c*), also within the *Kcnq1ot1* imprinted cluster but not *Ascl2* or *Slc22a18.* In addition, we observed disruption of maternally expressed *H19* but not paternally expressed gene 3 (*Peg3*). Placentae derived from the female offspring of High-concentration sires also exhibited suppression of *H19*, while Low-concentration placentae displayed an upregulation of *Peg3* (Figure 8B).

We next examined the expression of a cohort of genes encoding trophoblast-specific proteins and system A family amino acid transporters known to function downstream of the differentially expressed imprinted genes. Placentae derived from the male offspring of Low-concentration sires exhibited upregulation of solute carrier family 3 member 2 (*Slc3a2*) and trophoblast specific protein alpha (*Tpbpa*), while the female offspring displayed upregulation of both amino acid transporters (*Slc3a2* and *Slc28a2*) and *Tpbpa* (Figure 8C). We did not observe any alterations in these same genes in either male or female placenta derived from High-concentration sires. Finally, our previous studies examining alcohol-induced changes in epigenetic programming have identified altered expression of several mitochondrial-expressed transcripts and nuclear genes regulating oxidative phosphorylation and mammalian target of rapamycin (mTOR) signaling (Chang et al., 2021; Thomas et al., 2021). Similar to these previous studies, we identify suppression of these same candidate genes in placentae derived from the male and female offspring of Low-concentration sires but not the offspring of High-concentration males (Figure 8D). In summary, our studies reveal that Low-concentration EtOH exposures disrupt the regulation of select imprinted genes, which are crucial regulators of placental patterning and function, as well as elements regulating mitochondrial function.

### Discussion

Dose-response assessments represent a critical element of hazard characterization and typically define the threshold of exposure above which stressors cause adverse effects. Here, we employed a well-defined mouse model of voluntary EtOH exposure to examine the dose-response relationship of paternal drinking on the epigenetic programming of offspring fetoplacental growth. Our studies reveal a nonlinear biphasic or inverted J-shaped dose-response curve, where lower paternal exposures induce placental overgrowth and increased crown-rump length, while higher exposures trend towards placental growth restriction. The progressive, dose-dependent histological changes to the placental labyrinth layer we observe are similar to previous studies examining placental responses during fetal growth restriction (Coan et al., 2008). These observations reinforce the hypothesis that preconception paternal exposures transmit a stressor to the offspring that negatively impacts male placental development and function. Notably, we find that paternal low-dose EtOH exposures also transmit a memory to their offspring, inducing altered imprinted gene regulation and sex-specific impacts on placental histological organization and growth. To the best of our knowledge, this represents the first report describing the paternal epigenetic inheritance of a hormetic, biphasic dose-response in a mammalian system.

Our limited access model does not account for the EtOH preference of individual mice. Even after selecting higher-level drinkers, we still observed wide variation in individual preference, where mice assigned to a particular treatment group based on EtOH concentration achieved higher or lower doses than planned. Only by retrospectively adjusting for fluid consumption could we stratify our data and appropriately define dose-dependent effects, which revealed that lower dose exposures exert a potent influence on fetoplacental growth. Previously, we reported that the offspring of EtOH-exposed sires exhibit sex-specific changes in postnatal growth and glucose homeostasis (Chang et al., 2019a; Chang et al., 2019b). However, using vapor chambers to chronically expose males to EtOH, Rathod and colleagues did not observe any impacts on offspring growth or glucose metabolism (Rathod et al., 2020). Based on the dose-response data we now report, we suspect these discrepancies are due to the higher dose and longer duration of daily exposures employed in their study. Further, we suspect that sufficiently high doses of EtOH overwhelm and inhibit EtOH processing mechanisms (Pikkarainen, 1971), leading to the epigenetic programming of distinct, high-dose outcomes for various behavioral and growth measures.

In toxicology, the term hormesis describes circumstances where modest exposures to toxicants, hypoxia, or ionizing radiation induce stimulatory effects, while in contrast, sufficiently high exposures cause pathology (Leak et al., 2018). Notably, clinical studies have employed preemptive exposures to low-level stressors to exert beneficial outcomes in advance of high-level stressors or to stimulate recovery. The most notable of these examples is the role brief ischemic episodes have in improving the ability of tissues to recover from enduring ischemic attacks (Pan et al., 2016). Therefore, hormesis is a recognized element of human physiology, enhancing our abilities to recover from injury and adapt to environmental stressors (Leak et al., 2018). Accordingly, transmitting this adaptive information from parent to offspring would be evolutionarily advantageous.

Detoxification of EtOH is a capacity-limited process, where high exposures saturate the underlying enzymatic mechanisms and produce different toxic responses than lower exposures falling within detoxification capacity (Pikkarainen, 1971). For example, work examining the influence of alcohol on memory formation and adaptive responses in the expression of NMDA receptors revealed that low doses of EtOH challenge the brain to induce an adaptive state of neuroprotection, resulting in improved memory formation. In contrast, higher exposures exert toxic effects that inhibit memory formation (Kalev-Zylinska and During, 2007). Similar biphasic responses arise in the lymphatic and immune systems (Velazquez-Marrero et al., 2011; Lundgaard et al., 2018; Sureshchandra et al., 2019). Therefore, systemic challenges induced by low-dose EtOH exposures may stimulate the germline to transmit nongenomic information that enhances adaptation to this environmental toxicant.

Researchers suspect that EtOH-induced toxicity arises from reactive oxygen species or other oxidative stress-mediated processes (Herrera et al., 2003). Notably, multiple reactive oxygen species serve as a signaling mechanism for cellular systems to sense and initiate hormetic responses that reprogram gene expression toward a stable adaptive state (Cox et al., 2018; Leak et al., 2018). In worms, these changes alter chromatin structure, specifically histone H3, lysine four trimethylation (H3K4me3) in both the soma and the germline, enabling the transgenerational propagation of an adaptive state (Tauffenberger and Parker, 2014; Kishimoto et al., 2017). We have observed suppression of pathways regulating oxidative phosphorylation in a high-dose model of early gestational EtOH exposure and recently identified this same signature in male placentae sired by EtOH-exposed fathers (Chang et al., 2021; Thomas et al., 2021). Further, we have identified alcohol-induced increases in H3K4me3 in alcohol-exposed sperm (Bedi et al., 2022). Therefore, we speculate that low levels of oxidative stress program epigenetic changes in sperm, which suppress oxidative stress response pathways in the offspring, including the mTOR, EIf2, and Sirtuin signaling pathways. As hypoxia and oxidative stress stimulate growth and differentiation of the placenta (Pringle et al., 2010), suppressing these protective pathways may explain the enhanced growth we observe. Further, early gestational exposures to transient oxidative stress induce a similar repertoire of metabolic outcomes as those we identified in the male offspring of alcohol-exposed sires (Dimova et al., 2020). If our mouse studies directly translate to humans, gene-environment differences in oxidative stress responses may play an unappreciated role in the susceptibility to alcohol-induced teratogenesis and contribute to the wide variation in FASD phenotypes and prevalence observed in the clinics (Schaefer and Deere, 2011; McCarthy and Eberhart, 2014). Future studies are required to determine if H3K4me3 or other polycomb-mediated epigenetic changes participate in the intergenerational transmission of these effects.

Although these data are compelling, there are some limitations to our study. First, because we hypothesized that Low-concentration exposures would not impact offspring growth, we have not adequately explored the lower end of the dose-response curve to determine the minimum exposure level eliciting a response in placental growth. Further, we have a limited sample size of males in the higher range of the dose curve. Therefore, additional studies are necessary to model with effects of an occasional or infrequent drinker versus a chronic alcoholic. Second, to avoid the confounding impacts of stress, we subjected the males to limited handling and only measured plasma alcohol concentrations once. Therefore, we do not know if plasma alcohol levels consistently differed between treatments. Further, previous studies modeling binge drinking have revealed that using the Drinking in the Dark model, mice consume most (41%) alcohol during the first 15 min (Linsenbardt and Boehm, 2014). As we did not investigate consumption patterns within the 4-h exposure window, we do not know if differences in palatability between the different concentrations alter drinking behaviors, which could influence the duration and peak magnitude of blood plasma alcohol levels. Most studies modeling binge drinking do not employ low-dose exposures. Therefore, additional studies are required to determine if the Low-concentration treatment alters drinking behaviors and the impacts on plasma alcohol levels, and how this may relate to germline programming in the alcohol-exposed males.

Third, while the preconception period lasted 6 weeks, exposures continued during the breeding phase, with total preconception exposure periods ranging from 7 to 18 weeks. Although not the focus of this study, we did not observe any differences between placental phenotypes before or after 11 weeks, the mean exposure time. However, we do not know if longer exposures exert a cumulative impact on the sperm epigenome or if an exposure threshold was reached after the initial 6 weeks. Future studies will address this question, as well as the resilience of the sperm-inherited developmental program to recover after cessation of alcohol exposure. Fourth, we do not know if the observed increases in placental weight or fetal size in the Low-dose group are beneficial or exert long-term consequences on offspring growth and metabolic health. Furthermore, no studies have examined how preconception paternal alcohol exposures may interact with maternal exposures. Therefore, alcohol researchers should interpret these growth data cautiously and not presume these changes as beneficial. Fifth, our study only examines a single developmental time point, and the growth differences we observe may represent transient changes that disappear by birth. Finally, although maternal EtOH exposures also predominantly impact male placental weights (KwanCecilia et al., 2021), we have not identified any further experimental data to explain these sexually dimorphic outcomes. Future experiments will address these questions.

Due to the misconception that sperm only transmit genetic information, reproductive toxicologists have not adequately considered the impacts of paternal lifestyle and exposure history on offspring developmental outcomes. This is especially true for FASDs, where the fault is exclusively attributed to maternal exposures (Cook et al., 2016). Today, we recognize that a wide variety of preconception stressors, including cold, exercise, over and undernutrition, exposure to drugs of abuse, and intense psychological stress, all modify the paternally-inherited epigenetic program, with negative repercussions on offspring health (Champroux et al., 2018; Donkin and Barrès, 2018; Le Blévec et al., 2020). Our studies demonstrate that a lasting memory of low-dose alcohol exposures transmits to the offspring and provides a dose-response framework to further dissect the molecular mechanisms underlying the mammalian intergenerational inheritance of acquired traits.

## Data Availability

The raw data supporting the conclusion of this article will be made available by the authors, without undue reservation.
