# Supplementary material for: Paternal alcohol exposures program intergenerational hormetic effects on offspring fetoplacental growth
Source: Front Cell Dev Biol. 2022 Aug 11;10:930375. doi: 10.3389/fcell.2022.930375 (PMC9405020; doi:10.3389/fcell.2022.930375)
Supplement: Supplementary file 1 [file DataSheet1.PDF]

Supplemental Table 1.

Treatment	Average (weeks)	Range (weeks)	7	8	9	10	11	12	13	14	15	16	17	18
Control (22)	10.3	6 - 16	6		2	4	4	1	1	3		1		
Low (25)	10.2	7 - 13	4	2	2	3	6	5	3					
Medium (15)	11.2	7 - 18	3	1	1		2	1	1	4		1		1
High (18)	11.8	6 - 17	3		2	3	1	1	2		4		2	
**Total**			13	3	7	10	13	8	7	7	4	2	2	1

Main Breeding Phase

Supplemental Table 1. The number of litters conceived during each week of the breeding phase. The number of litters sired by each treatment group during the breeding phase from weeks seven to fifteen. Red lines indicate the mean time required to generate all examined litters. Note: one Control, two Medium-, and two High-concentration litters were sired between weeks sixteen and eighteen.

Supplemental Table 2.

Treatment	Started	Culled
Control	22	0
Low	25	0
Medium	15	1
High	28	10

Supplemental Table 2. The number of males eliminated from each treatment group after falling below the weekly fluid consumption threshold of 0.08 g/g/week for three consecutive weeks.

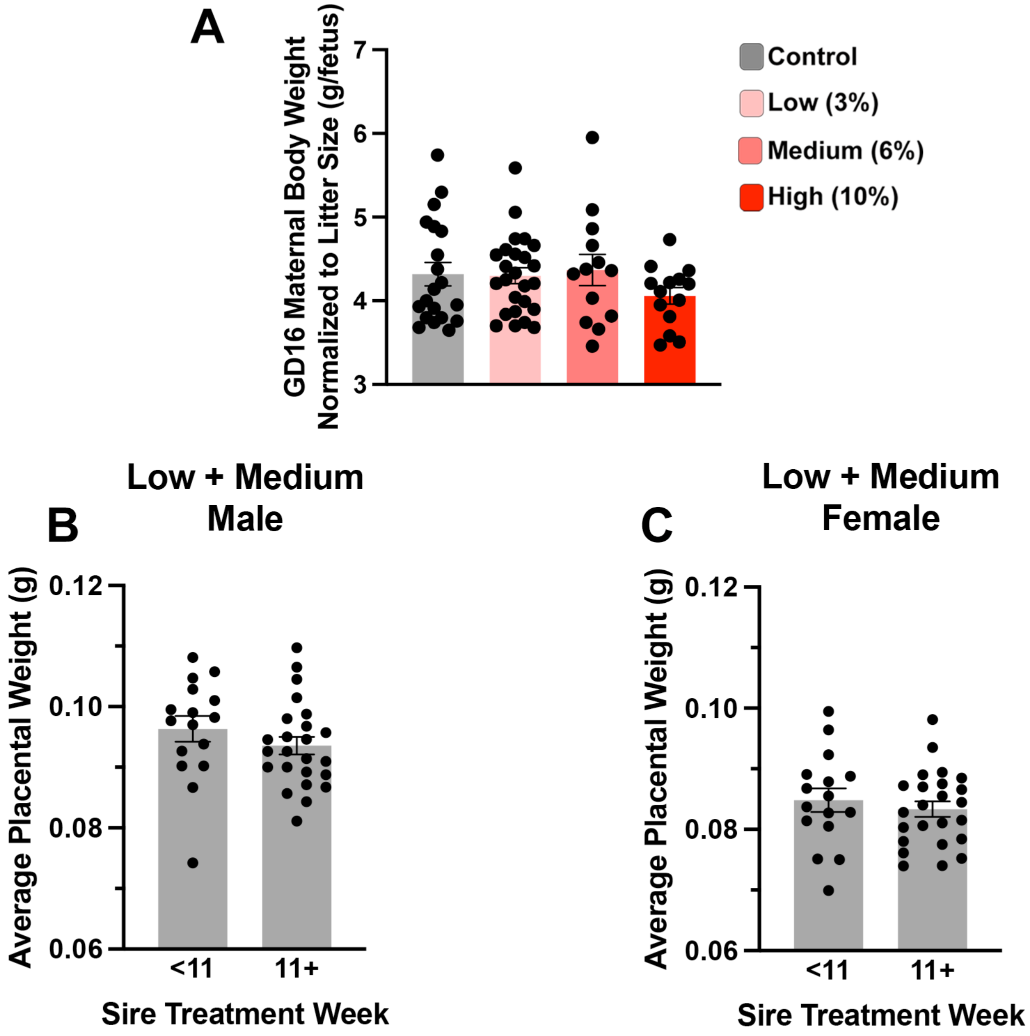

Supplemental Figure 1. A) Comparison of gestational day 16.5 dam weights normalized to litter size. We normalized dam weights to litter size by calculating the (g) dam weight per fetus, then used a one-way ANOVA to compare measures between treatment groups (20 Control, 25 Low, 13 Medium, 14 High). Comparison of B) male and C) female offspring placental weights before and after eleven weeks of continuous ethanol exposure. We combined the litter average placental weights for the Low- and Medium-concentration treatments and separately compared male and female offspring before and after 11 weeks exposure, which was the median exposure duration for the combined dataset. We compared measures using an unpaired t-test (16 litters before week 11 and 24 litters after week 11).

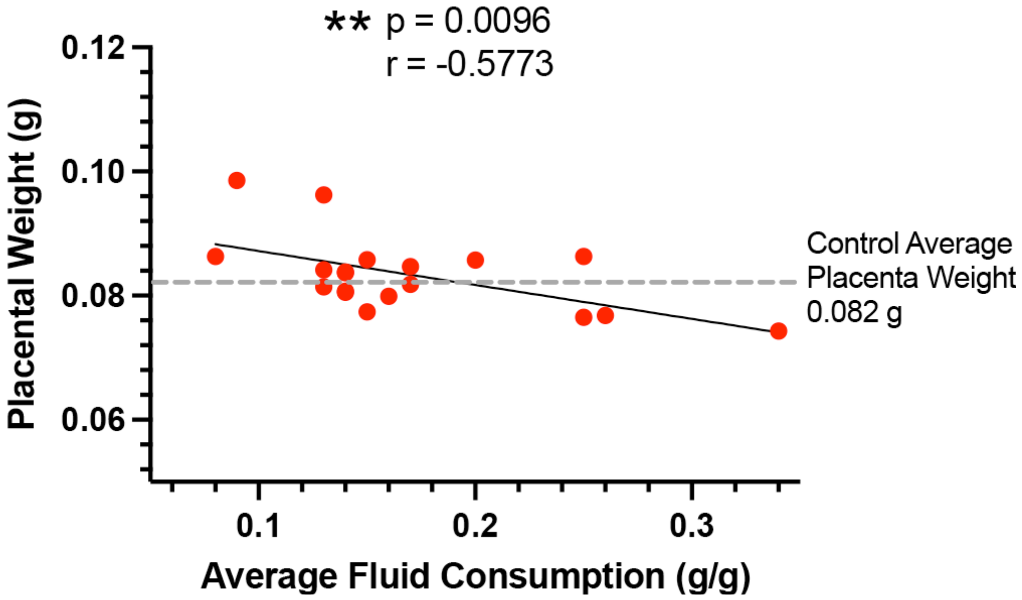

Supplemental Figure 2. Pearson correlation analysis contrasting female offspring litter average placental weights and average paternal fluid consumption across the High-concentration treatment group (n=18 litters). Error bars represent the standard error of the mean, ** P < 0.01.

Supplemental Table 3.

	**1. Pearson Correlation**				**2. Regression Analysis**		
**Figure**	**r**	**R squared**	**P-value**	**Summary**	**Model**	**Goodness of Fit - R squared**	**Runs test, Deviation from model**
4C	**-0.6041**	**0.3649**	**0.0062**	**	Linear (*Straight line)*	0.3649	Cannot perform (same x-value)
5A	**-0.5479**	**0.3002**	**0.0021**	**	Polynomial: Fourth Order	0.4034	***ns***
5B	**-0.5492**	**0.3017**	**0.0317**	*	Sine wave	0.3799	***ns***
5E	-0.2969	0.08817	0.1178	***ns***	Polynomial: Fourth Order	0.1094	***ns***
5F	-0.2009	0.04034	0.2961	***ns***	Polynomial: Fourth Order	0.1205	***ns***
6F	**-0.6983**	**0.4877**	**0.0079**	**	Linear (*Straight line)*	0.4877	***ns***
Decidua Female (data not shown)	-0.5178	0.2681	0.1028	***ns***	Sine wave	0.4940	***ns***
6G	**0.6409**	**0.4108**	**0.0183**	*	Linear (*Straight line)*	0.7269	***ns***
Labyrinth Female (data not shown)	0.4095	0.1677	0.2110	***ns***	Sigmoidal *(Bottom unconstrained)*	0.7580	***ns***

Supplemental Table 3. Supporting information for Pearson correlation and regression analyses. We performed Pearson correlations to assess overall trends (y-value increasing or decreasing with x-value), then we fit the data to an appropriate linear or nonlinear regression model. We determined the goodness of fit, R squared, but since this value is discretionary and can be interpreted to various degrees across fields, we employed a Runs test to establish if the data deviated from the model. The abbreviation "*ns*" (*not significant*) indicates that the data does not significantly deviate from the model and thus that the implemented model is valid. * $P < 0.05$, ** $P < 0.01$.
